# GC-TOF-MS-Based Non-Targeted Metabolomic Analysis of Differential Metabolites in Chinese Ultra-Long-Term Industrially Fermented Kohlrabi and Their Associated Metabolic Pathways

**DOI:** 10.3390/metabo12100991

**Published:** 2022-10-19

**Authors:** Xin Nie, Hongfan Chen, Lu Xiang, Yulin Zhang, Dayu Liu, Zhiping Zhao

**Affiliations:** 1 College of Food Science and Technology, Sichuan Tourism University, Chengdu 610100, China; 2 College of Food and Biological Engineering, Chengdu University, Chengdu 610106, China; 3 Sichuan Key Laboratory of Solid State Fermentation Resource Utilization, Yibin Univerisity, Yibin 644000, China

**Keywords:** kohlrabi, metabolomics, GC-TOF-MS, differential metabolites, metabolic pathway

## Abstract

Fermented kohlrabi is a very popular side dish in China. Chinese kohlrabies industrially fermented for 0 years (0Y), 5 years (5Y), and 10 years (10Y) were employed and analyzed by non-targeted metabolomics based on GC-TOF-MS, and the differential metabolites were screened using multivariate statistical analysis techniques, including principal component analysis (PCA) and orthogonal partial least squares discrimination analysis (OPLS-DA). The results showed that 47, 38, and 33 differential metabolites were identified in the three treatment groups of 0Y and 5Y (A1), 0Y and 10Y (A2), and 5Y and 10Y (A3), respectively (VIP > 1, *p* < 0.05). The metabolites were mainly carbohydrates, amino acids, and organic acids. Furthermore, 13 differential metabolites were screened from the three groups, including L-glutamic acid, L-aspartic acid, *γ*-aminobutyric acid, and other compounds. Four metabolic pathways termed alanine, aspartate, and glutamate metabolism, arginine biosynthesis, arginine and proline metabolism, and glycolysis/gluconeogenesis were the most significant pathways correlated with the differential metabolites, as analyzed according to the Kyoto Encyclopedia of Genes and Genomes (KEGG). The odors for the three ultra-long-term industrially fermented kohlrabies were significantly different, as detected by E-nose. The present work describes the changes in metabolites between different ultra-long-term industrially fermented kohlrabies and the associated metabolic pathways, providing a theoretical basis for the targeted regulation of characteristic metabolite biosynthesis in Chinese fermented kohlrabi.

## 1. Introduction

Kohlrabi (*Brassica juncea* var. *megarrhiza* Tsen et Lee) is a root-type variant of mustard, belonging to the cruciferous *Brassica* annual herb, which is rich in vitamins, fiber, mineral elements, and other nutrients [1]. Kohlrabi originated in China as a cross-pollinating crop with small flower organs and is mainly used as a raw material to make Chinese pickles. Kohlrabi is widely distributed in China and mainly grown in Sichuan, Yunnan, Guizhou, Hunan, Hubei, Jiangsu, and Zhejiang Provinces. The crop is usually sown from mid-to-late August to early September and harvested from December to January of the next year. The suitable growth temperature for kohlrabi is 15–20 °C. In addition to its edible value, kohlrabi also has certain medicinal values. It has been suggested that regular consumption of *Brassica* plants can effectively reduce the incidence of colon cancer and rectal cancer, as well as cardiovascular and degenerative diseases, immune dysfunction, and age-related macular degeneration [2]. Raw kohlrabi has a strong mustard spicy taste and thus it is not suitable as a side dish before fermentation. The mustard spicy taste is generally removed by fermentation and fermented kohlrabi becomes crisp and tender, with a rich aroma and a delicious taste, making it a very popular side dish in Chinese cuisine. Yang et al. [3] analyzed the effects of high-hydrostatic-pressure treatment and heat treatment on the texture of kohlrabi, and suggested that high-hydrostatic-pressure treatment could better maintain the textural properties of kohlrabi. The flavor of fermented kohlrabi is closely correlated with production processes and raw materials. Jiang et al. [4] found that raw materials could produce significantly different flavors of kimchi. By using headspace solid-phase microextraction combined with gas chromatography–mass spectrometry, Zhang and co-workers [5] determined and analyzed the types and contents of flavor substances in mustard cabbage using traditional and modern fermentation processes. A total of 78 flavor substances were detected, including 16 esters, 11 aldehydes, 18 alcohols, 8 ketones, 6 acids, 5 olefins, 7 phenols, and 7 others. The main different flavor components between the two different fermented mustard cabbages were allyl isothiocyanate, ethyl acetate, 3-butyronitrile, phenol, ethanol, and acrolein.

Metabolomics is a qualitative and quantitative technique used to analyze changes in small-molecule metabolites with a relative molecular mass less than 1000 in organisms and their changes after stimulation and interference [6]. According to experimental purposes, metabolomics is normally divided into targeted metabolomics and non-targeted metabolomics. In recent years, metabolomics has been widely used in drug evaluation, disease diagnosis and treatment, plant stress-resistance research, and functional food development. Nuclear magnetic resonance spectroscopy, gas chromatography–mass spectrometry, gas chromatography–time-of-flight mass spectrometry (GC-TOF-MS), and liquid chromatography–mass spectrometry hyphenated techniques are the commonly used techniques in metabolomics. GC-TOF-MS exhibits more advantages, such as high mass resolution and accuracy, high scanning speed, and good sensitivity [7], and has been widely used to quantify and identify metabolites in various fields of research, such as carbohydrates, sugar alcohols, organic acids, and amino acids [8]. Metabolomics can also be employed to evaluate the flavor and nutritional value of food [9]. The development of dishes based on fermented kohlrabi depends on the metabolite contents.

To date, research on the metabolomics of Chinese ultra-long-term industrially fermented kohlrabi has not been reported in the literature, and the metabolic pathways closely related to the metabolites in Chinese ultra-long-term industrially fermented kohlrabi are poorly understood. In this study, non-targeted metabolomics of GC-TOF-MS combined with multivariate statistical analysis methods were used to measure and analyze the related metabolites of Chinese ultra-long-term (0-year, 5-year, and 10-year) industrially fermented kohlrabi, for the first time, and to elucidate their associated metabolic pathways. The 10-year fermented kohlrabi is normally used for soup, while, the 5-year fermented kohlrabi is suitable for Chinese braised dishes. The three different kohlrabies possess significantly different sensory properties. The experimental results will provide some theoretical support for differential metabolite synthesis in industrially fermented kohlrabi and the product development of functional kohlrabi.

## 2. Materials and Methods

### 2.1. Production of Ultra-Long-Term Industrially Fermented Kohlrabi

The kohlrabies were cultivated by local farmers in Chengjia Town (latitude 29 degrees 39′ north and longitude 104 degrees 21′ east) in the same area of Zigong City, China, and were of the same variety (Queyecai). After maturity, the kohlrabies were harvested and washed with water. Then, the washed kohlrabies were air-dried outside for about 30 days. After air-drying, the kohlrabies were transferred into fermentation tanks. Eight percent (*w*/*w*) salt (upper layer 60%, middle layer 30%, and lower layer 10%) was added to the kohlrabies and they were pickled for 5 days. Then, 5% (*w*/*w*) salt (upper layer 10%, middle layer 30%, and lower layer 60%) was added to the once-pickled kohlrabies and they were pickled for a further 4 days. Finally, all the fermentation tanks were completely sealed, and the pickled kohlrabies were fermented for 5 years (5Y) and 10 years (10Y). The kohlrabies were fermented in 200 kg tanks. There were 1000–1600 kohlrabies per 200 kg tank, depending on the size of the kohlrabies. All tested samples were vacuum-packaged in aluminum foil bags and stored at -80 °C for further analysis.

### 2.2. Physicochemical Analysis

pH values were measured with a pH meter (PHS-3C, Shanghai Sanxin Instrument, Shanghai, China). Color measurements for redness (a*), yellowness (b*), and brightness (L*) were performed using a colorimeter (RC-10, Konica Minolta, Tokyo, Japan). The total acid contents, reducing sugar contents, and soluble protein contents were respectively determined by the acid–base titration method [10], the direct titration of the alkaline copper tartrate solution method [11], and the spectrophotometry method [12]. The salt contents were determined by Dought’s method [13].

### 2.3. Extraction of Metabolites from Ultra-Long-Term Industrially Fermented Kohlrabi

Extraction of metabolites from ultra-long-term industrially fermented kohlrabies was performed according to the method of Oliver and Tobias [14]. Fifty milligrams of kohlrabi was transferred into a 2 mL Eppendorf tube and 0.5 mL of solution (acetonitrile:isopropanol:water (3:3:2, *v*/*v*/*v*)) was added to each tube. Then, four zirconium beads with sizes of 2 mm were placed in the tube and the kohlrabi was ground using a high-throughput tissue grinder (Scientz Biotechnology, SCIENTZ-48, Ningbo, China). The sample was centrifuged at 12,000 rpm for 2 min after grinding, and 2 mL of the supernatant was transferred into a clean Eppendorf tube, which was subsequently dried in a vacuum concentrator. The dried powder was resuspended in 80 μL of 20 mg/mL methoxamine pyridine and incubated at 60 °C for 60 min. Finally, 100 μL of BSTFA-TMCS (*v*/*v*, 99:1) was added and incubated at 70 °C for 90 min after 30 s of vortexing. The resuspension was centrifuged at 14,000 rpm (Cence, H1850-R, Changsha, China) for 3 min, and 100 μL of the supernatant was transferred into a detection bottle.

### 2.4. Detection of Metabolites from Ultra-Long-Term Industrially Fermented Kohlrabi by GC-TOF-MS

GC-TOF-MS was performed as described in a previous study [15]. A DB-5MS capillary column (30 m × 250 µm i.d., 0.25 µm film thickness; Agilent J & W Scientific, Folsom, CA, USA) was used for gas chromatography. Derivatized species were separated at a constant flow of helium at 1 mL/min, and 1 µL of the sample was injected through the autosampler in a split ratio of 1:10. The inlet temperature was 280 °C, and the transfer line and ion source temperatures were 320 °C and 230 °C, respectively. The heating program was: initial temperature 50 °C, lasting 0.5 min, increased to 320 °C at a rate of 15 °C/min and held at 320 °C for 9 min. A full-scan method was used, with a scan rate of 10 spec/s, an electron energy of −70 V, and a solvent delay of 3 min.

### 2.5. GC-TOF-MS Data Preprocessing and Analysis

The obtained GC-TOF-MS raw data were converted into a common format by Analysis Base File Converter software and subsequently pre-processed by MSDIAL software. Differential metabolites were characterized according to molecular weight (molecular weight error < 30 ppm). Fragmentation information obtained in MS/MS mode was further matched and annotated in the HMDB, Metlin, Massbank, LipMaps, mzclound, and BioNovoGene (Suzhou, China) databases. The matrix containing metabolite names and peak intensities was processed according to missing-value imputation and normalization and subsequently imported into SIMCA (version 14.1) for PCA and OPLS-DA analysis.

### 2.6. Metabolic Pathway Analysis

Various public databases were searched, including the KEGG and PubChem databases, to further validate the details of the identified metabolites. The screened differential metabolites were mapped to MetaboAnalyst 5.0 (www.metaboanalyst.ca, accessed on 10 March 2022) for related metabolic pathway analysis.

### 2.7. E-Nose Analysis

Electronic-nose analysis was performed using a Fox 4000 Sensory Array Fingerprint Analyzer (Alpha M.O.S., Toulouse, France), which contains 18 metal oxide semiconductors. A description for all 18 sensors was given in a previous study [16]. For E-nose analysis, 0.5 g of fermented kohlrabies was ground and transferred into a 10 mL headspace bottle. The balance time was 5 min at 70 °C. The temperature of the injection module was 70 °C. The injection speed was 500 μL/s, and the injection period was 1 s. The data-acquisition time was 120 s. The detection time was 180 s. A PCA plot of the E-nose was employed to further analyze all the samples. Five parallel experiments were performed for every sample.

### 2.8. Statistical Analysis

Student’s *t*-tests were performed on metabolites with VIPs > 1 in the OPLS-DA model using IBM SPSS Statistics (version 24), and the significance level was set at *p* < 0.05. The fold change for each metabolite was calculated by EXCEL (version 2016). After log2 processing, the differential metabolites were defined with |log2 FC| > 1.1, VIP > 1, and *p* < 0.05 as the standard.

## 3. Results

### 3.1. Physicochemical Analysis

The physicochemical analyses of the three ultra-long-term industrially fermented kohlrabies are listed in Table 1. The pH and reducing sugar contents gradually decreased and the total acid contents thus constantly increased. The protein contents became lower and lower, which was possibly the result of proteolysis catalyzed by proteases. The colors of the industrially fermented kohlrabies changed significantly, as shown in Figure S1. The color of the 5Y fermented kohlrabi was nearly red, and its L* value was smaller than that of the 0Y fermented kohlrabi but bigger than that of the 10Y fermented kohlrabi.

### 3.2. PCA Analysis for the Metabolomics of Ultra-Long-Term Industrially Fermented Kohlrabies

**Figure 1:**
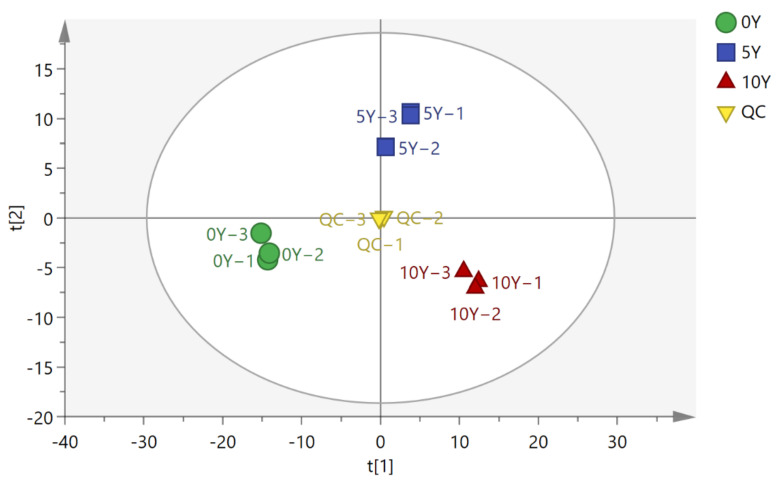
The scatter plots of the PCA model for the 0Y, 5Y, and 10Y fermented kohlrabi samples representing 0-year, 5-year and 10-year ultra-long-term industrially fermented kohlrabies, respectively.

Based on GC-TOF-MS combined with multivariate statistical methods, the differential metabolites of the three kohlrabies were explored. Twenty microliters of each extract from the three different kohlrabies were mixed together and the obtained mixture was used as a quality control (QC). QC is often required to obtain high-quality metabolomic data, as revealed in Figure 1. The aggregation degree of the QC was better than that of the 0Y, 5Y, and 10Y ultra-long-term fermented kohlrabies, indicating that the experimental data were reliable. All data for the 0Y, 5Y, and 10Y kohlrabies were distributed in different regions, indicating significant differences in the metabolites among the three different groups of fermented kohlrabi. R^2^X and Q^2^ were the main parameters for judging the PCA model, R^2^X = 0.72 > 0.5 and Q^2^ = 0.536, suggesting that the PCA model could well interpret the significant differences among the differential metabolites in the three kohlrabies.

### 3.3. OPLA-DA Analysis of Metabolites from Three Different Kohlrabies

**Figure 2:**
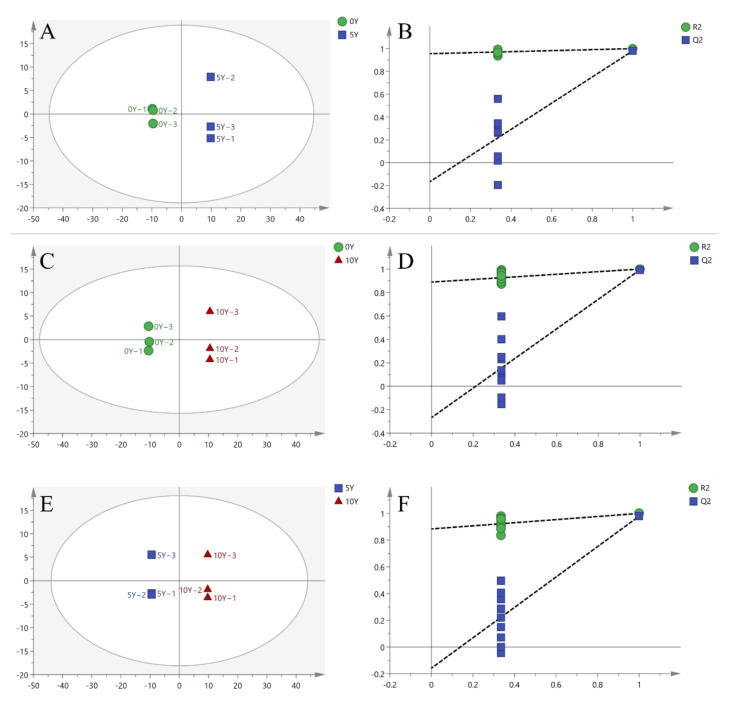
Scatter plots and permutation tests of the OPLS-DA models for fermented kohlrabi samples. (**A**,**C**,**E**) represent the OPLS-DA analyses for A1 (0Y–5Y), A2 (0Y–10Y), and A3 (5Y–10Y), while (**B**,**D**,**F**) represent permutation tests for A1 (0Y–5Y), A2 (0Y–10Y), and A3 (5Y–10Y).

For further analysis of the three treatment groups (A1, A2, and A3), OPLS-DA analysis was performed and obtained good separation, as shown in Figure 2. Figure 2A,C,E indicated the OPLS-DA score maps for the three kohlrabi groups, respectively. It was obvious that all groups were significantly separated, implying that the significant differences in the types and contents of metabolites from the three kohlrabies were caused by the different ultra-long-term fermentation periods. The OPLS-DA model parameters listed in Table 2 could explain and predict the differences between every two kohlrabi metabolites. In order to further verify the model was reliable, 200-loop-iteration permutation tests were performed, as can be seen in Figure 2B,D,F. All the intersections for the Q^2^ regression line and Y-axis were on the negative half-axis, indicating that the OPLS-DA model was stable and reliable. Moreover, no overfitting phenomenon was observed.

### 3.4. Screening and Identification of Differential Metabolites from the Three Industrially Fermented Kohlrabies

**Figure 3:**
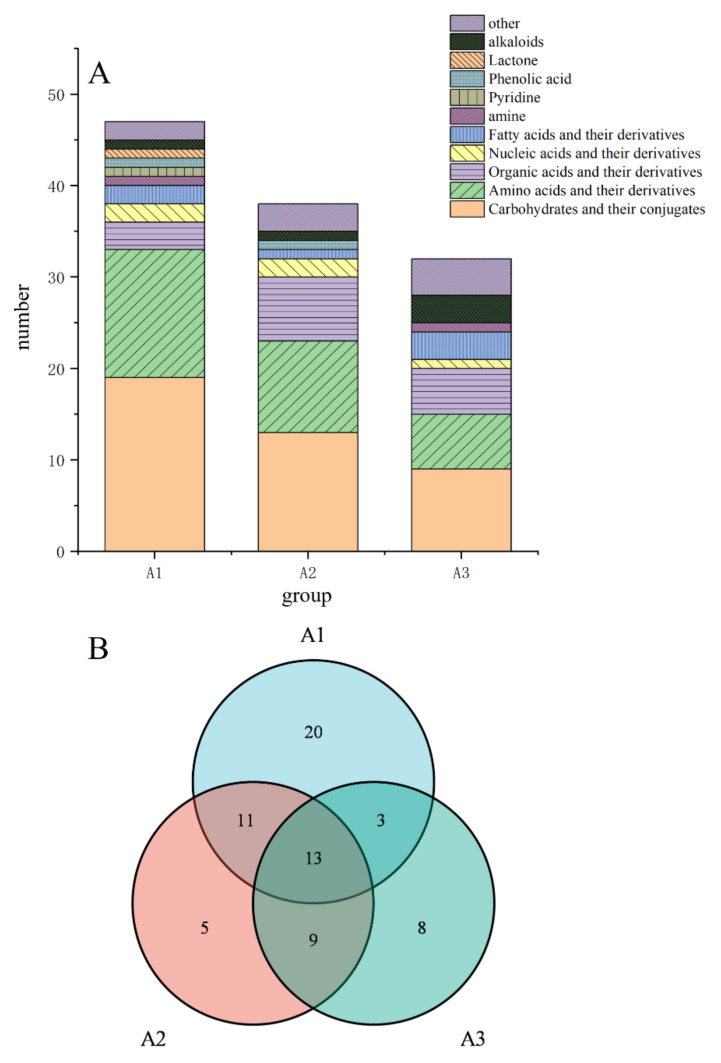
Classification information (**A**) and Venn diagram (**B**) for the differential metabolites in industrially fermented kohlrabi. (A1: Chinese kohlrabies industrially fermented for 0 years and 5 years, A2: Chinese kohlrabies industrially fermented for 0 years and 10 years, A3: Chinese kohlrabies industrially fermented for 5 years and 10 years.)

In this study, VIP > 1 and *p* < 0.05 were used as the screening standards for differential metabolites from ultra-long-term industrially fermented kohlrabi. Based on the set standards, 47, 38, and 33 differential metabolites were respectively screened from group A1, A2, and A3, as shown in Figure 3A. Carbohydrates and carbohydrate conjugates, amino acids and their derivatives, and organic acids and their derivatives were the main differential metabolites in the three industrially fermented kohlrabies. Additionally, more organic acids were produced with the extension of fermentation time. The differential metabolites of the three industrially fermented kohlrabies were analyzed using a Venn diagram, as shown in Figure 3B. Thirteen major differential metabolites were identified from the three industrially fermented kohlrabies, including six carbohydrates and carbohydrate conjugates (2-amino-3,4,5,6-tetrahydroxyhexanal, 3,6-anhydrogalactose, *α*-D-quinolulose, *β*-lactose, D-(+)-cellobiose, methylpyran glycosides), one amino acid and one derivative (DL-aspartic acid, 2-amino-3-methylsuccinic acid), one nucleic acid derivative (uracil), one fatty acid derivative (lactitol), one alkaloid (leucine), and two other metabolites (2,3-butanediol, galactosylglycerol).

### 3.5. Heat Map Analysis for Differential Metabolites

**Figure 4:**
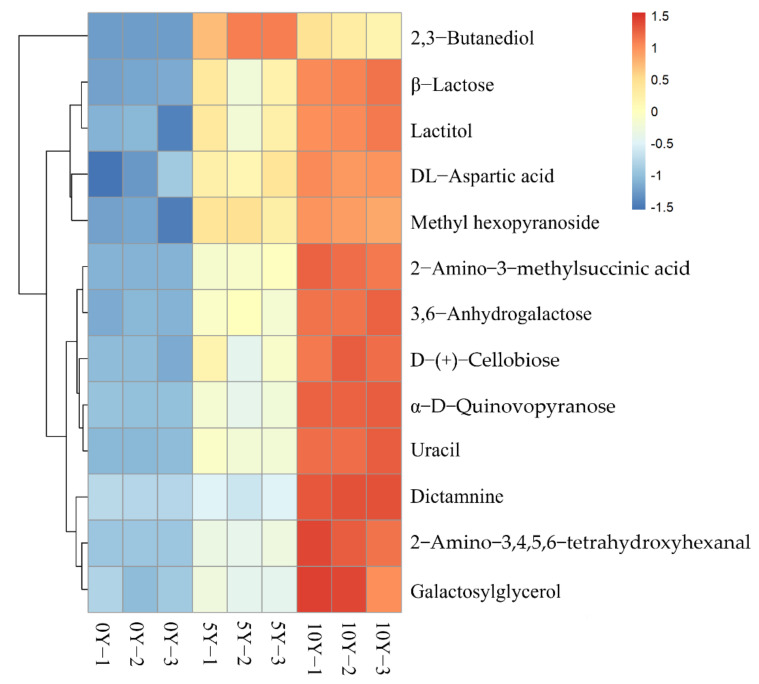
Hierarchical clustering analysis heat map of the main differential metabolites in ultra-long-term industrially fermented kohlrabi samples.

In order to better evaluate the metabolic patterns of the main differential metabolites in industrially fermented kohlrabi, the relative contents of the 13 differential metabolites in the industrially fermented kohlrabies were analyzed by hierarchical clustering analysis and displayed in the form of a heat map, as shown in Figure 4. It was obvious that all the main differential metabolites increased with the extension of fermentation, except 2,3-butanediol. The main differential metabolites with higher contents in 10Y kohlrabi were *β*-lactose, lactitol, DL-aspartic acid, methylhexoside, 2-amino-3-methylsuccinic acid, 3,6-anhydrogalactose, D-(+)-cellobiose, *α*-D-quinovopirose, uracil, dictamnine, 2-amino-3,4,5,6-tetrahydroxyhexanal, and galactosylglycerol. However, the main differential metabolite with a higher content in 5Y kohlrabi was 2,3-butanediol.

### 3.6. Metabolic Pathway Analysis for Differential Metabolites in Ultra-Long-Term Industrially Fermented Kohlrabi

**Figure 5:**
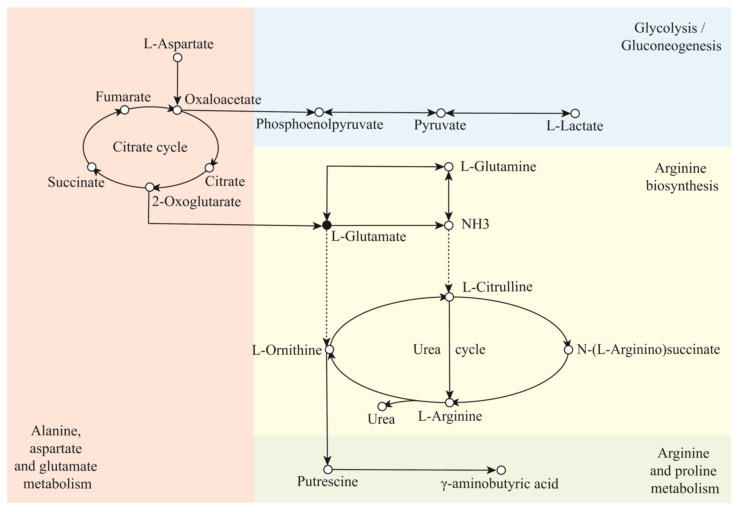
Integrated analysis diagram of the main metabolic pathways in ultra-long-term industrially fermented kohlrabi.

The identified differential metabolites were imported into the KEGG database to obtain perturbed metabolic pathway information. Consequently, four main pathways were enriched, termed alanine, aspartate and glutamate metabolism (impact = 0.82), arginine biosynthesis (impact = 0.26), arginine and proline metabolism (impact = 0.24), and glycolysis/gluconeogenesis (impact = 0.13), as shown in Figure 5. Alanine and aspartic acid and glutamic acid were metabolized with L-aspartic acid as a raw material, which was converted into oxaloacetate under the catalysis of α-amino acid oxidase. 2-oxoglutaric acid was generated from oxaloacetate through the citric acid cycle, which was subsequently converted into L-glutamic acid, catalyzed by L-glutamic acid synthase. L-glutamic acid acted as a raw material in the arginine biosynthetic pathway and was catalyzed by glutamate dehydrogenase (NADP+) to generate NH_3_, which was subsequently converted into L-citrulline under the catalysis of carbamoyl phosphate synthase, acetylornithine transcarbamylase, and acetylornithinease [17]. The metabolism of arginine and proline employed L-glutamic acid as raw material, which produced L-ornithine under the catalysis of glutamate kinase, glutamate semialdehyde dehydrogenase, and ornithine aminotransferase. The L-ornithine was catalyzed by ornithine decarboxylase to produce putrescine, which was subsequently converted into *γ*-aminobutyric acid through putrescine-pyruvate aminotransferase and the catalysis of other enzymes. Oxaloacetate generated by the citric acid cycle was used as the precursor for the glycolysis/gluconeogenesis pathway, which was subsequently converted into phosphoenolpyruvate under the catalysis of phosphoenolpyruvate carboxykinase. Phosphoenolpyruvate further generated pyruvate by phosphoenolpyruvate carboxyl synthase, which was finally converted into lactic acid, catalyzed by lactate dehydrogenase.

### 3.7. E-Nose Analysis

**Figure 6:**
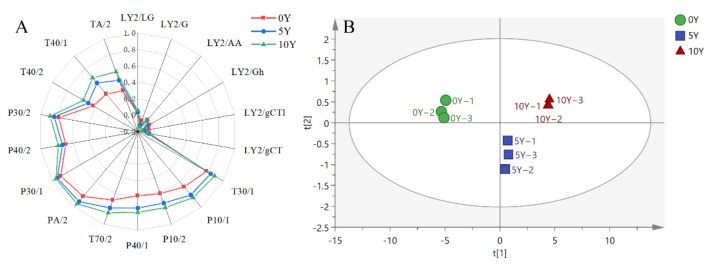
E-nose analysis. (**A**) Radar graph of the three different industrially fermented kohlrabies. Blue, red, and black indicate 0Y, 5Y, and 10Y industrially fermented kohlrabies, respectively. (**B**) PCA plot of the three different industrially fermented kohlrabies.

As analyzed by E-nose, the differences in nitrogen oxides, amines, sulfides, and acetone among the three different fermented kohlrabies were not significant, as revealed in Figure 6A. However, significant differences in polar substances, non-polar substances (hydrocarbons, ammonia, chlorine), aromatics (toluene, xylene), amines, and chlorines were observed in the three different industrially fermented kohlrabies. With the extension of fermentation, the sensor signals became stronger, suggesting that the contents of the related compounds increased, which results agree well with the changes in the metabolites.

PCA is the method normally used to generate principal component variables to eliminate correlations between original feature variables. As shown in Figure 6B, the three different fermented kohlrabies were presented in a PCA spatial distribution map. The first two components (PC1 and PC2 were 96.70% and 2.08%, respectively) explained nearly 99% of the total variance, indicating that the PCA model can better reflect the overall information for the samples [18]. The contribution of PC1 was much higher than that of PC2, implying that the farther the samples were on the horizontal axis, the greater the difference in smell. The spatial regions of these samples showed that the odors for the 0Y, 5Y, and 10Y industrially fermented kohlrabies were significantly different.

## 4. Discussion

During fermentation, the carbohydrates were metabolized by microorganisms to generate acids; consequently, the total acid contents increased and the pH and reducing sugar contents decreased [19]. Generally, the growth of most spoilage microorganisms will be inhibited when the pH is lower than 3.8 and the total acid is more than 0.5%, resulting in the safety of fermented vegetables. The flavor and metabolites will be significantly changed with the extension of fermentation. It has been well demonstrated that microorganisms continuously utilize the substrates in kohlrabi through enzymatic reactions to maintain their normal physiological functions [20], leading to the hydrolysis of proteins and fats to generate amino acids and fatty acids. As shown in Figure S2, during the fermentation process, the contents of organic acids and their derivatives, amino acids and their derivatives, and carbohydrates and carbohydrate conjugates increased with the extension of fermentation. It has been suggested that the contents of organic acids, amino acids, aldehydes, and ketones would significantly increase in fermented wild mustard greens with the extension of fermentation, which would promote the formation of unique flavors [21]. Organic acids are important factors in the flavors and aromas of fermented foods and play an irreplaceable role in the formation of flavor and overall sourness during the fermentation process. Lactic acid is the most important acid-tasting substance in Chinese paocai, which is produced by the anaerobic fermentation and metabolism of lactic acid bacteria. Lactic acid is an important flavor substance in fermented vegetables, which provides the fresh, sour taste to products. With the extension of fermentation time, the contents of lactic acid in the fermented kohlrabies increased. During the fermentation process of Chinese suancai, the pH kept decreasing and the total titratable acidity thus kept increasing [22]. Organic acids are considered the intermediate products of many life activities in plants and play important roles in regulating plant life activities. For example, salicylic acid and benzene-ring-containing carboxylic acids and their derivatives could systemically induce and produce resistance in a variety of plants [23]. Similarly, malic acid could increase tolerance to water stress in different plants [24]. On the other hand, organic acids could prevent the accumulation of reactive oxygen species in cells and give plants the ability to avoid oxidative stress [25] through maintaining intracellular pH, potential, and redox balance [26].

Amino acids are a class of important biologically active organic compounds. As shown in Figure S2, among the 69 differential metabolites detected, 10 free amino acids can be divided into 4 groups: sweet amino acids (L-ornithine), bitter amino acids (L-valine, L-isoleucine, L-leucine, L-methionine), tasty amino acids (DL-aspartic acid, L-glutamate), and tasteless amino acids (L-glutamine, DL-homoserine, *γ*-aminobutyric acid). During fermentation, sweet amino acid contents first increased and then decreased. Except for L-methionine, all the other bitter amino acids showed increasing trends in the A1 (0Y–5Y) group. However, their levels stabilized in the A3 (5Y–10Y) group. The fresh amino acids showed an upward trend during fermentation, which possibly explained why the 10Y fermented kohlrabi was suitable for soup. Besides their important contributions to the taste of fermented vegetables [27], free amino acids are also important flavor precursors. Some free amino acids produced by protein hydrolysis can be converted to small molecular compounds, such as aldehydes, ketones, and hydrocarbons, through a series of reactions, such as deamination and decarboxylation, which have important impacts on the taste of fermented food. The contents of free amino acids in kohlrabi changed significantly during the ultra-long-term fermentation, indicating that the fermentation period was closely related to product taste. Generally, the free amino acids generally showed a trend of first rising and then slightly declining during fermentation, which was consistent with a previous study [28], possibly due to the conversion of free amino acids into volatile flavor substances by microorganisms. The contents of amino acids were generally affected by a variety of factors. It has been reported that wheat-grain free amino acids were affected by soil type and irrigation water salinity levels [29]. However, free amino acids in tomato plants were highly dependent on nitrogen formation and concentration in the rooting medium. Li and co-workers demonstrated that the contents of free amino acids in mustard were significantly affected by different light treatments. Furthermore, the contents of free amino acids in sprouted mustard greens were significantly higher than in mustard seeds. Meanwhile, light treatment promoted the synthesis of free amino acids in mustard [30]. The community structure of microorganisms and their physiological metabolism have a significant impact on amino acid contents in fermentation systems. Lactic acid bacteria decompose proteins into various free amino acids under the catalysis of secreted polypeptide enzymes [31]. Zhao et al. suggested that *Lactobacillus plantarum* and *Lactobacillus brucei* could significantly increase levels of glutamic acid, glycine, and gamma-aminobutyric acid in Chinese paocai, as measured by GC-MS [32]. Amino acids are used as the main biological macromolecular materials for building organisms and play important roles in plants, including building cells and repairing tissue damage, enhancing plant metabolism, and improving plant stress resistance. Amino acids could affect the growth of plants by influencing the coordinated distribution of nitrogen sources and nitrogen in plants. Increasing leaf nitrogen allocation could significantly enhance leaf nitrogen fixation and nitrogen-use efficiency by photosynthesis [33].

Amino acids also play a positive role in the stress response of many plants as a kind of regulator. For example, proline, tryptophan, leucine, isoleucine, and valine were considered to be related to drought stress in plants [34]. However, L-tryptophan, L-glutamic acid, and L-phenylalanine played important roles in salt stress in Tibetan highland barley [35]. It is well known that glutamate and homocysteine participate in cadmium stress in mustard leaves [36]. On the other hand, amino acids have effects on plant flowering under stress conditions. For example, aspartic acid, glutamic acid, alanine, glycine, and serine could promote plant flowering, while cysteine, threonine, and phenylalanine inhibit plant flowering [37].

Carbohydrates participate in the physiological reaction processes of cells in plants and can also be used as structural components and metabolic components in living organisms [38]. For example, carbohydrates act as important components of signaling pathways that connect and construct networks of pathways to control metabolic responses in plants. It has been suggested that carbohydrates induce stress responses and increase plant resistance to abiotic stress [39].

Secondary metabolites are a class of organic compounds with complex structures which are synthesized through a series of metabolic pathways using a large number of primary metabolites as precursors, including alkaloids, pigments, antibiotics, phenolic acids, and other substances [40]. Benzoate was the only differential metabolite among phenolic acids detected in ultra-long-term industrially fermented kohlrabi. Under enzymatic reactions, phosphoenolpyruvate and erythrose-4-phosphate generate phenylalanine through the shikimate pathway, which is subsequently converted into trans-cinnamic acid, benzoic acid, salicylic acid, and 4-hydroxychalcone, and thus enters the flavonoid metabolic pathway [41]. It has been suggested that alkaloids play important anti-tumor and analgesic roles, among which are a class of nitrogen-containing basic organic compounds, mainly including isoquinolines, pyridines, etc. [42]. In this study, two differential metabolites of alkaloids, dictamnine and 2-hydroxypyridine, were detected in ultra-long-term industrially fermented kohlrabi. Furthermore, the contents of the two alkaloids were dramatically increased during the fermentation process. Dictamnine is a small-molecule alkaloid with various biological activities, which is normally synthesized through the shikimic acid pathway and can be extracted from various plants. On the other hand, dictamnine has many beneficial biological activities, such as anti-platelet aggregation, anti-hypertension, antibacterial, anti-mitotic, and anti-fungal activities [43].

The change in 2,3-butanediol observed in this work was consistent with a previous study in which, with the extension of fermentation, the content of 2,3-butanediol in Chinese suancai first increased and then decreased [44]. Carbohydrates could generate acetoin through a series of enzymatic reactions, which is subsequently converted into by-products, such as 2,3-butanediol and lactic acid, under the action of acetoin reductase in a reversible way [45]. Xiong et al. indicated that during the fermentation process of Chinese paocai, lactic acid bacteria consumed a large amount of sucrose and glucose to produce lactic acid. The accumulation of lactic acid resulted in a decrease in the content of 2,3-butanediol [46], which finding possibly supports our result that the content of 2,3-butanediol in 10Y ultra-long-term industrially fermented kohlrabi was lower than that in 5Y ultra-long-term industrially fermented kohlrabi. 2,3-Butanediol was the common differential metabolite among the three ultra-long-term fermented kohlrabies, and its contents were significantly different among the three kinds of fermented kohlrabies, as revealed in Figure 4, such that it might be used as an important biomarker to distinguish the different ultra-long-term fermented kohlrabies.

The 5Y and 10Y kohlrabies are respectively used for making soup and Chinese braised dishes, which possibly correlates with the metabolites in the two different kohlrabies. The contents of dictamnine, galactosylglycerol, linoleate, L-Rhamnose, *β*-Lactose, (9Z,12Z,15Z)-Octadecatrienoic acid, galacturonan, lactitol, L-Isoleucine, and threonate continuously increased, with the highest level in 10Y kohlrabi (Figure S2), among which the dictamnine, galactosylglycerol, L-rhamnose, and (9Z,12Z,15Z)-octadecatrienoic acid possess the function of inhibiting cancer cell proliferation [47]. Moreover, linoleate, galacturonan, and hexadecanoic acid exhibit antibacterial and anti-inflammatory activities [48]. Lactitol and L-isoleucine are used to treat liver disease and hyperinsulinism [49,50]. Threonate has a certain therapeutic effect on Alzheimer’s disease [51]. L-rhamnose, lactitol, lactic acid, linoleate, hexadecanoic acid, and pentose are flavor substances, with sweet, sour, fatty, and smoky-sweet-salty-coffee tastes, respectively, playing very important roles in flavor formation in 10Y kohlrabi. However, the levels of D-tagatose, L-leucine, tranexamic acid, D-(−)-fructose and 2,3-butanediol were higher in 5Y kohlrabi than in the 0Y and 10Y kohlrabies (Figure S2), among which D-tagatose, L-leucine, and tranexamic acid have the function of promoting weight loss, neuron regeneration, and hemostasis, respectively [52,53,54], while D-(−)-fructose brings a clean sweet taste and 2,3-butanediol gives the fruity and creamy aroma which contributes to the flavor of 5Y kohlrabi.

The colors of the three different kohlrabies were significantly different, as can be seen in Figure S1. Tyrosine was detected in all the three different kohlrabies and its contents gradually decreased, with the lowest level in 10Y kohlrabi (Figure S2). Tyrosine can be converted into dopa under the catalysis of tyrosinase, which can generate melanin through oxidation [55] and probably has a potential impact on the color of kohlrabi.

## 5. Conclusions

In the present study, GC-TOF-MS combined with multivariate statistical analysis was used to determine the differential metabolites in Chinese ultra-long-term industrially fermented kohlrabies that underwent fermentation for different periods and to clarify their associated metabolic pathways. There were 47, 38, and 33 differential metabolites in the three groups of kohlrabi industrially fermented for 0 year and 5 years, 0 year and 10 years, and 5 years and 10 years, respectively (VIP > 1, *p* < 0.05). The differential metabolites were mainly carbohydrates, amino acids, and organic acids, and there were 13 common differential metabolites, including L-glutamic acid, L-aspartic acid, *γ*-aminobutyric acid, etc. Through KEGG metabolic pathway analysis, four metabolic pathways termed alanine, aspartate and glutamate metabolism, arginine biosynthesis, arginine and proline metabolism, and glycolysis/gluconeogenesis were finally identified. The correlation with differential metabolites was the most significant, which may provide some theoretical support for subsequent research on fermented kohlrabi. Traditionally fermented kohlrabi undergoes complex biochemical reactions during the fermentation process. The metabolic pathways are normally complex, and the metabolites are numerous. Moreover, the odors of the three ultra-long-term industrially fermented kohlrabies were significantly different. Further in-depth analysis of the changes in the metabolites and metabolome of kohlrabi during fermentation is required.

## Figures and Tables

**Table 1:** Changes in physicochemical properties of ultra-long-term industrially fermented kohlrabies.

	0Y	5Y	10Y
pH	6.26 ± 0.01 ^a^	3.59 ± 0.04 ^b^	3.47 ± 0.02 ^c^
Total acid (%)	0.26 ± 0.02 ^a^	0.88 ± 0.06 ^b^	0.91 ± 0.01 ^b^
Reducing sugar (g/100 g)	5.47 ± 0.1 ^a^	2.47 ± 0.01 ^b^	2.05 ± 0.04 ^c^
Protein (g/100 g)	2.68 ± 0.15 ^a^	1.87 ± 0.13 ^b^	1.24 ± 0.13 ^c^
Salt content (%)	12.06 ± 0.2 ^a^	12.64 ± 0.14 ^b^	12.66 ± 0.16 ^b^
Brightness (L*)	56.31 ± 1.2 ^a^	31.54 ± 1.08 ^b^	22.03 ± 0.52 ^b^
Redness (a*)	3.47 ± 0.17 ^a^	10.4 ± 0.19 ^b^	5.75 ± 0.29 ^c^
Yellowness (b*)	20.78 ± 0.65 ^a^	14.34 ± 0.4 ^b^	9.64 ± 0.16 ^c^

Different superscript letters in the same row indicate significant differences (*p* < 0.05).

**Table 2:** Parameters of the OPLS-DA models for the industrially fermented kohlrabi samples.

Group	R^2^X	R^2^Y	Q^2^
A1 (0Y–5Y)	0.719	0.999	0.980
A2 (0Y–10Y)	0.775	1.000	0.994
A3 (5Y–10Y)	0.688	1.000	0.979

## Data Availability

The data supporting the results of this study are included in the present article.

## Supplementary Materials

The following supporting information can be downloaded at: https://www.mdpi.com/article/10.3390/metabo12100991/s1, Figure S1: The appearance of the Chinese fermented kohlrabi. A, B, and C, respectively, indicated 0Y, 5Y, and 10Y fermented kohlrabi; Figure S2: Clustering heat map of differential metabolites of industrially fermented kohlrabi with different ultra-long-term fermentation periods.
