# Supplementary material for: GC-TOF-MS-Based Non-Targeted Metabolomic Analysis of Differential Metabolites in Chinese Ultra-Long-Term Industrially Fermented Kohlrabi and Their Associated Metabolic Pathways
Source: Metabolites. 2022 Oct 19;12(10):991. doi: 10.3390/metabo12100991 (PMC9610423; doi:10.3390/metabo12100991)
Supplement: Supplementary file 1 [file metabolites-12-00991-s001.zip › Supplementary Figures.pdf]

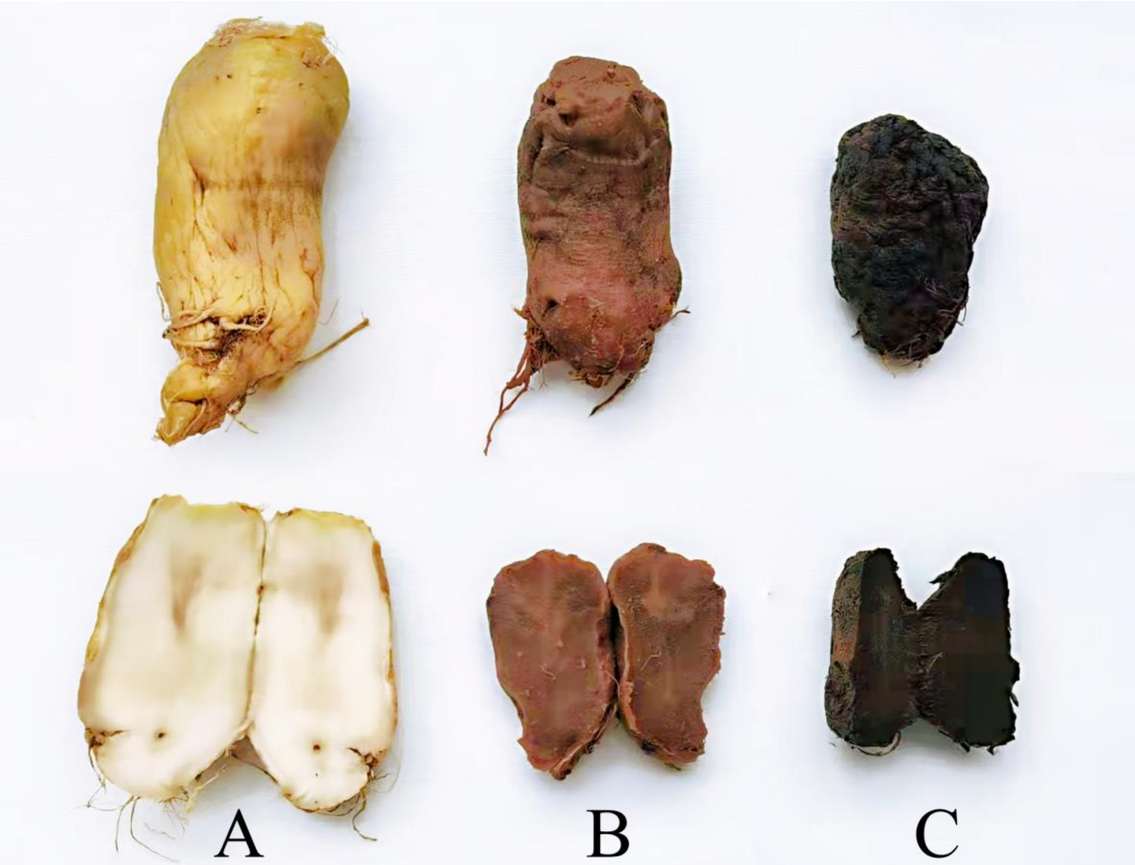

Figure S1. The appearance of the Chinese fermented kohlrabi, A, B and C respectively indicated 0Y, 5Y and 10Y fermented kohlrabi.

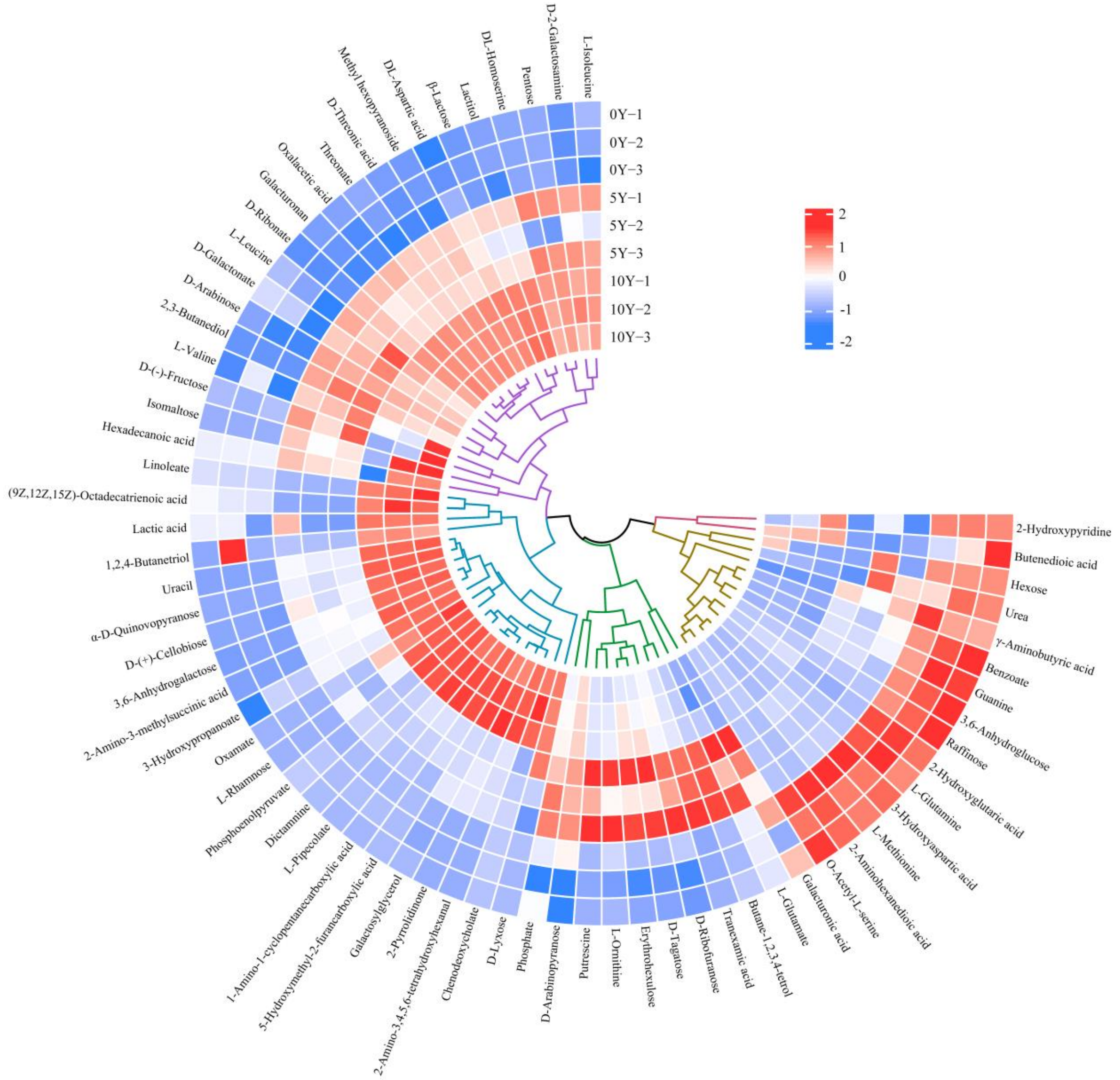

Figure S2. Clustering heat map of differential metabolites of industrial fermented kohlrabi with different ultra-long-terms fermentation.
